# Louseborne Relapsing Fever in Young Migrants, Sicily, Italy, July–September 2015

**DOI:** 10.3201/eid2201.151580

**Published:** 2016-01

**Authors:** Alessandra Ciervo, Fabiola Mancini, Francesca di Bernardo, Anna Giammanco, Giustina Vitale, Piera Dones, Teresa Fasciana, Pasquale Quartaro, Giovanni Mazzola, Giovanni Rezza

**Affiliations:** Istituto Superiore di Sanità, Rome, Italy (A. Ciervo, F. Mancini, G. Rezza);; Azienda di Rilievo Nazionale ed Alta Specializzazione Civico Di Cristina e Benfratelli, Palermo, Italy (F. di Bernardo, P. Dones);; University of Palermo, Palermo (A. Giammanco, T. Fasciana);; Azienda Ospedaliera Universitaria Policlinico, Palermo (G. Vitale, P. Quartaro, G. Mazzola)

**Keywords:** louseborne relapsing fever, body louse, Borrelia recurrentis, bacteria, migrants, refugees, refugee camps, louseborne infections, vector-borne infections, zoonoses, Libya, Somalia, Sicily, Italy

**To the Editor:** During the early 20th century, at the end of World War I, and during World War II, louseborne relapsing fever (LBRF) caused by *Borrelia recurrentis* was a major public health problem, especially in eastern Europe and northern Africa (*1**,**2*). Currently, poor living conditions, famine, war, and refugee camps are major risk factors for epidemics of LBRF in resource-poor countries, such as those in the Horn of Africa (*3**,**4*).

Increased migration from resource-poor countries and war/violence create new routes for spread of vectorborne diseases. Recently, several cases of LBRF have been reported among asylum seekers from Eritrea in the Netherlands, Switzerland, and Germany (*5**–**8*). All of these asylum seekers had been in refugee camps in Libya or Italy. We report 3 cases of LBRF in migrants from Somalia to refugee camps in Sicily, Italy.

Patient 1 was a 13-old-boy from Somalia who arrived in Palermo, Italy, on July 11, 2015, after traveling though Libya. He was admitted to G. Di Cristina Hospital in Palermo 5 days after arrival because of high fever, headache, and general malaise, which developed 2 days after arrival. The patient had skin lesions on his fingers and legs and a conjunctival infection. He had thrombocytopenia (79,000 platelets/μL [reference range 150 platelets/μL−400 platelets/μL]), creatine phosphokinase level 967 mg/L [reference range 0.001 mg/L–0.10 mg/L], aspartate aminotransferase level 30 U/L (reference value 37 U/L), and alanine aminotransferase level 21 U/L (reference value 41 U/L). He was given ceftriaxone (2 g/d) and intravenous hydration. His conditions worsened ≈10 hours after treatment: high fever (temperature 40°C), chills, and profuse sweating (Jarish-Herxheimer reaction). The patient recovered after 15 days of treatment with ceftriaxone. A Giemsa-stained blood smear was negative for *Plasmodium* spp. but showed large numbers of spirochetes. Serologic screening results for *B. burgdorferi* were negative.

Patient 2 was a 17-old-boy from Somalia who arrived in Lampedusa, Italy, on August 27, 2015, after traveling through Libya. Fever and artromyalgia developed 6 days after his arrival, and he was admitted to Hospital Paolo Giaccone in Palermo. Blood analyses showed increased levels of aminotransferases, thrombocytopenia (69,000 platelets/μL), and mild anemia (hemoglobin level 94 g/L [reference range 130 g/L–160 g/L]). A blood smear was negative for *Plasmodium* spp. but positive for spirochetes. Serologic screening results were negative for malaria, leptospirosis, infection with *Rickettsia conorii*, and dengue. An ELISA result was positive for *B. burgdorferi*, and a Western blot result was positive for *Borrelia* spp. proteins p10, p41, and OspC. The patient recovered after treatment with doxycycline (100 mg/d) and ceftriaxone (2 g/d) for 10 days.

Patient 3 was a 17-year-old boy from Somalia who arrived in Trapani, Italy, on September 4, 2015. He reported that he stayed for 5 months in Libya before arriving in Italy. Fever, artromyalgia, severe dehydration, renal failure, and mental confusion developed 3 days after his arrival, and he was admitted to Hospital Paolo Giaccone. He had severe thrombocytopenia (4,000 platelets/μL); mild anemia (hemoglobin level 88 g/L); increased levels of aminotransferases (aspartate aminotransferase 282 U/L, alanine aminotransferase 489 U/L), lactate dehydrogenase (1,041 U/L [reference range 105 U/L−333 U/L]), d-dimer (6,311 ng/mL [reference range 10 ng/mL–250 ng/mL]), C-reactive protein (237.8 mg/dL [reference range 0 mg/dL–10 mg/dL]), and creatinine (2.6 mg/dL [reference range 0.6 mg/dL–1.2 mg/dL]); and azotemia (blood urea nitrogen level 150 mg/dL [reference range 7 mg/dL–20 mg/dL]). A blood smear was negative for *Plasmodium* spp., but a Giemsa-stained thick blood smear was positive for spirochetes. Serologic screening results were negative for malaria, leptospirosis, infection with *B. burgdorferi*, and dengue. The patient recovered after treatment with doxycycline (100 mg/d) and ceftriaxone (2 g/d) for 10 days.

DNA was extracted from blood specimens from the 3 patients and used for molecular identification and characterization of the etiologic agent of LBRF. We used a species-specific real-time PCR for *B. recurrentis* and *B. duttonii*, which targeted an internal region of the *recN* gene. Multispacer sequence typing of the 16S rRNA gene was used for bacterial identification and genotyping (*9**,**10*). All blood samples were positive for *B. recurrentis* by real-time PCR. Multispacer sequences showed 100% identity with sequences of *B. recurrentis* reference strain A1 (GenBank accession no. CP000993) for isolates from all patients.

We report 3 patients in Italy with LBRF who migrated from Somalia. These patients arrived in Italy after traveling in several countries in Africa and crossing the Mediterranean Sea. The patients did not associate with each other during travel, and the place where they were infected is unknown. However, because they came from a disease-endemic country, they probably had been infested with body lice and were infected with *B*. *recurrentis* in Somalia or other neighboring countries.

Because the 3 cases we observed might indicate that more migrants and refugees are infected, LBRF should be considered an emerging disease among migrants and refugees. Diagnostic suspicion of LBRF should lead to early diagnosis among refugees from the Horn of Africa and in persons in migrant camps. Furthermore, improved public health measures and hygiene must be implemented for persons in refugee or migrant camps.

## References

[1] Raoult D, Roux V. The body louse as a vector of reemerging human diseases. Clin Infect Dis. 1999;29:888–911. 10.1086/520454 10589908

[2] Cutler SJ, Abdissa A, Trape JF. New concepts for the old challenge of African relapsing fever borreliosis. Clin Microbiol Infect. 2009;15:400–6. 10.1111/j.1469-0691.2009.02819.x 19489922

[3] Cutler SJ. Possibilities for relapsing fever reemergence. Emerg Infect Dis. 2006;12:369–74. 10.3201/eid1203.050899 16704771 PMC3291445

[4] European Centre for Disease Control and Prevention (ECDC). Louse-borne relapsing fever; factsheet for health professionals. Stockholm: ECDC [cited 2015 Sep 25]. http://ecdc.europa.eu/en/healthtopics/emerging_and_vector-borne_diseases/vector-borne_diseases/louse-borne-relapsing-fever/Pages/Factsheet-for-health-professionals.aspx

[5] Wilting KR, Stienstra Y, Sinha B, Braks M, Cornish D, Grundmann H. Louse-borne relapsing fever (*Borrelia recurrentis*) in asylum seekers from Eritrea, the Netherlands, July 2015. Euro Surveill. 2015;20:21196. 26250069 10.2807/1560-7917.es2015.20.30.21196

[6] Goldenberger D, Claas GJ, Bloch-Infanger C, Breidthardt T, Suter B, Martínez M, Louse-borne relapsing fever (*Borrelia recurrentis*) in an Eritrean refugee arriving in Switzerland, August 2015. Euro Surveill. 2015;20:21204. 26290486

[7] Loescher T, Wieser A, Fingerele V. Louse-borne relapsing fever—Germany: asylum seekers, ex East Africa. ProMed. 2015 Sep 3 [cited 2015 Sep 28]. http://www.promedmail.org archive no. 3620174.3.

[8] Frank C, Hendrik Wilking H. Louse-borne relapsing fever—Germany (02): asylum seekers. ProMed. 2015 Sep 11 [cited 2015 Sep 28]. http://www.promedmail.org archive no. 3638819.11.

[9] Elbir H, Henry M, Diatta G, Mediannikov O, Sokhna C, Tall A, Multiplex real-time PCR diagnostic of relapsing fevers in Africa. PLoS Negl Trop Dis. 2013;7:e2042. 10.1371/journal.pntd.0002042 23390560 PMC3561136

[10] Elbir H, Gimenez G, Sokhna C, Bilcha KD, Ali J, Barker SC, Multispacer sequence typing relapsing fever Borreliae in Africa. PLoS Negl Trop Dis. 2012;6:e1652. 10.1371/journal.pntd.0001652 PMC3367985 22679518

